# Effects of Psychological Interventions on Depression Symptoms in Oncology Patients: A Systematic Review

**DOI:** 10.15388/Amed.2026.33.1.5

**Published:** 2026-07-22

**Authors:** Ignas Lapeikis, Ieva Armonaitė, Vakarė Baranauskytė, Irina Banienė

**Affiliations:** 1 Lithuanian University of Health Sciences, Faculty of Medicine, Kaunas, Lithuania; 2 Lithuanian University of Health Sciences, Faculty of Medicine, Kaunas, Lithuania; 3 Lithuanian University of Health Sciences, Faculty of Medicine, Kaunas, Lithuania; 4 Lithuanian University of Health Sciences, Department of Health Psychology, Kaunas, Lithuania

**Keywords:** cancer, depression, psycho-oncology, psychological interventions, cognitive behavioural therapy, mindfulness, systematic review, vėžys, depresija, psichoonkologija, psichologinės intervencijos, kognityvinė elgesio terapija, dėmesingo įsisąmoninimo praktikos, sisteminė apžvalga

## Abstract

**Background:**

Depression is one of the most common psychological comorbidities among cancer patients and contributes substantially to a reduced quality of life and impaired treatment adherence. A range of psychosocial interventions have been proposed to address depressive symptoms in oncology, but evidence across intervention types remains heterogeneous.

**Aim:**

To evaluate the effectiveness of psychological interventions in reducing depressive symptoms among cancer patients.

**Methods:**

A systematic search of *PubMed, ScienceDirect, Web of Science*, and *Clinical Key* databases was conducted for studies published between 2016 and 2026. Randomized controlled trials and controlled intervention studies assessing psychological interventions for depressive symptoms in adult cancer patients were included. Data on the study design, patient characteristics, intervention type, depression assessment scales, and outcomes were extracted. Due to heterogeneity in interventions and outcome measures, the findings were synthesised narratively.

**Results:**

Twenty studies met the inclusion criteria. Interventions included cognitive behavioural therapy (CBT), mindfulness-based interventions (MBIs), mindfulness-based stress reduction (MBSR), mindfulness-based cognitive therapy (MBCT), and positive psychology approaches. Most studies reported significant reductions in depressive symptoms measured by using validated scales such as PHQ-9, HADS, BDI, and SDS. CBT- and mindfulness-based approaches demonstrated the most consistent improvements, although several trials reported no significant differences compared with control groups.

**Conclusions:**

Psychological interventions, and particularly CBT- and mindfulness-based approaches, are associated with reductions in depressive symptoms among cancer patients. However, substantial heterogeneity in the intervention design and outcome reporting limits direct comparison across studies. Further large-scale randomized trials using standardised outcome measures are needed to establish optimal psychosocial treatment strategies in oncology.

## Abstract (it)

**Įvadas:**

Depresija yra viena iš dažniausių vėžiu sergančių pacientų psichologinių gretutinių ligų ir reikšmingai prisideda prie prastesnės gyvenimo kokybės bei blogesnio gydymo laikymosi. Onkologijoje depresijos simptomams mažinti taikomos įvairios psichosocialinės intervencijos, tačiau skirtingų intervencijų rūšių įrodymai išlieka heterogeniški.

**Tikslas:**

Įvertinti psichologinių intervencijų veiksmingumą mažinant vėžiu sergančių pacientų depresijos simptomus.

**Metodai:**

Atlikta sisteminė paieška „PubMed“, „ScienceDirect“, „Web of Science“ ir „ClinicalKey“ duomenų bazėse, įtraukiant 2016–2026 m. publikuotus tyrimus. Įtraukti atsitiktinių imčių kontroliuojami tyrimai ir kontroliuojami intervenciniai tyrimai, kuriuose vertintos psichologinės intervencijos, skirtos suaugusių vėžiu sergančių pacientų depresijos simptomams mažinti. Buvo renkami duomenys apie tyrimo dizainą, pacientų charakteristikas, intervencijos tipą, depresijos vertinimo skales ir rezultatus. Dėl intervencijų ir baigčių vertinimo priemonių heterogeniškumo rezultatai apibendrinti naratyviniu būdu.

**Rezultatai:**

Įtraukimo kriterijus atitiko dvidešimt tyrimų. Intervencijos apėmė kognityvinę elgesio terapiją (KET), dėmesingo įsisąmoninimo principais grįstas intervencijas, dėmesingu įsisąmoninimu grįstą streso mažinimą, dėmesingu įsisąmoninimu grįstą kognityvinę terapiją ir pozityviosios psichologijos metodus. Daugumoje tyrimų nustatytas reikšmingas depresijos simptomų sumažėjimas, vertintas validuotomis skalėmis, tokiomis kaip PHQ-9, HADS, BDI ir SDS. KET ir dėmesingo įsisąmoninimo metodais grįstos intervencijos parodė nuosekliausią pagerėjimą, nors keliuose tyrimuose reikšmingų skirtumų, palyginti su kontrolinėmis grupėmis, nenustatyta.

**Išvados:**

Psichologinės intervencijos, ypač KET ir dėmesingo įsisąmoninimo metodais grįstos intervencijos, yra susijusios su vėžiu sergančių pacientų depresijos simptomų sumažėjimu. Tačiau reikšmingas intervencijų dizaino ir rezultatų pateikimo heterogeniškumas riboja tiesioginį tyrimų palyginimą. Reikia tolesnių didelės apimties atsitiktinių imčių tyrimų, taikant standartizuotas baigčių vertinimo priemones, siekiant nustatyti optimalias psichosocialinio gydymo strategijas onkologijoje.

## Introduction

Cancer is one of the leading causes of premature death globally, particularly among individuals aged 30–70 years. For every 10 persons who die prematurely due to non-communicable diseases, three die of cancer [1]. Worldwide, the 5-year prevalence of all cancers is estimated at 50.5 million people [2].

Mental comorbidities, particularly depression, are common among individuals diagnosed with cancer [3]. A meta-analysis of 94 interview-based studies reported that depression affects approximately 16% of patients at any stage of treatment [4]. In breast cancer, pooled prevalence rates are even higher, with depressive symptoms observed in 20–24% of patients [5]. Psychological symptoms in oncology populations are frequently under-recognized and under-treated, as emotional distress is often interpreted as a normal reaction to diagnosis or attributed to physical disease-related symptoms [2].

Beyond its impact on the quality of life, depression may influence oncological outcomes [6]. A meta-analysis including more than 2.6 million individuals demonstrated that depression and anxiety were associated with a 13% increased risk of cancer incidence and a 21–24% higher cancer-specific mortality [7]. In breast cancer specifically, depression was associated with a 30% increase in all-cause mortality and a 24% increase in recurrence [8]. While causality remains debated, these associations are clinically significant.

The proposed biological mechanisms include dysregulation of the hypothalamic–pituitary–adrenal axis, immune suppression, and elevated inflammatory cytokines such as interleukin-6 and C-reactive protein [9,10]. Psychological distress is also correlated with poorer adherence to anticancer treatments and delays in seeking care, thus further compromising prognosis [11].

Psychosocial and psychiatric interventions, including cognitive behavioural therapy, mindfulness-based stress reduction, and structured exercise programs, have demonstrated efficacy in reducing depressive symptoms among cancer patients [12–14]. Accordingly, this systematic review aims to evaluate and synthesize existing evidence on the effectiveness of stress-reducing psychological interventions in reducing depression symptoms among adult patients with distinct types of cancer. In contrast to prior meta-analyses that have often been limited to specific cancer types due to methodological constraints [15–17], this systematic review allows inclusion of more diverse oncology populations and provides a broader synthesis of available evidence.

### Objectives

To analyse the characteristics and severity of depression symptoms among cancer patients, considering disease diagnosis, stage, treatment modality, and sociodemographic factors.

To analyse different psychological intervention types and their effectiveness in reducing the severity of depression symptoms among cancer patients.

## Materials and Methods

### Search Strategy

This systematic review was conducted in accordance with the *Preferred Reporting Items for Systematic Reviews and Meta-Analyses* (PRISMA) guidelines.

A comprehensive literature search was performed in four major medical databases: *PubMed, ScienceDirect, Web of Science*, and *Clinical Key*. The search covered the period from January 1, 2016, to February 1, 2026, to ensure that only contemporary evidence was included. The search strategy employed a combination of keywords and Boolean operators, including primary keywords like *psychological interventions, depression, psychological distress, cancer patients, oncology*. Boolean combinations: (*psychological interventions* OR *psychotherapy* OR *cognitive behavioural therapy* OR *mindfulness*) AND (*depression* OR *psychological distress*) AND (*cancer patients* OR *oncology*). Relevant Medical Subject Headings (MeSH) terms were also applied to enhance search sensitivity.

### Eligibility Criteria

The inclusion criteria for this study were: 1) studies that focused on adult cancer patients aged 18 years or older, 2) published in English, and with open access to full-text versions being provided, 3) randomized controlled trials, cohort studies, and case-control studies evaluating psychological interventions and their effects on depression in oncology patients were considered. The study’s exclusion criteria were as follows: 1) case reports, conference abstracts, editorials, or commentaries, 2) studies lacking clearly defined depression outcomes, 3) articles in languages other than English, 4) literature reviews or meta-analyses, 5) studies with inaccessible full text.

The selection process involved an initial screening of titles and abstracts in order to remove duplicates and clearly irrelevant studies. Four reviewers independently assessed the full texts of potentially eligible articles, and any disagreements were resolved through discussion until consensus was reached.

### Data Extraction and Management

For each included study, information was systematically extracted, including the authors and publication year, the study design, aim, population (age, cancer type and stage, and treatment modalities), the type of psychological intervention, psychological outcomes (depression levels and the assessment tools applied), as well as the main findings and the risk of bias.

To assess the methodological quality, the *Newcastle–Ottawa Scale* (*NOS*) was used for cohort, and case-control studies and Cochrane RoB 2.0 tool for RCT. Quality assessments were conducted independently by four reviewers, and discrepancies were resolved through discussion. Data synthesis was primarily narrative, by summarizing the key findings across the included studies.

Before the first screening phase, 478 records were excluded because of other reasons (titles did not contain any relevant keywords related to psychological intervention, depression, or oncology), and 38 duplicates were removed. The remaining 355 abstracts were independently reviewed by four investigators. After assessing their relevance and methodological suitability, 51 articles were excluded because they were in languages other than English, and 183 were removed due to an inaccessible full text. Out of 121 full-text articles, 57 were removed due to not mentioning depression outcomes, and 44 were excluded as literature reviews or meta-analyses.

**Figure 1:**
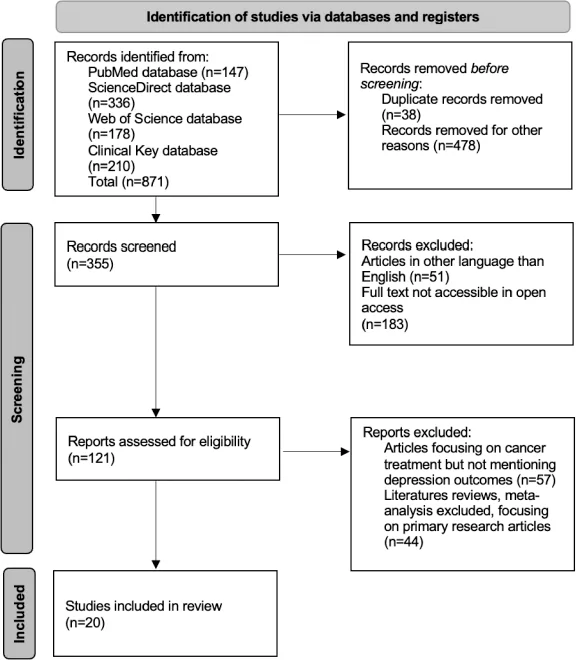
PRISMA flow diagram. 871 articles were identified through database searching. After removal of duplicates, title and abstract screening and further full-text assessment, 20 articles were included in the analysis

Ultimately, 20 studies met all inclusion criteria and were included in the final analysis. The detailed selection process is illustrated in **Figure 1**.

## Results

Study-level data were extracted into a structured spreadsheet by using a pre-specified extraction framework (**Table 1**). The extracted items included the study design, aim, sample characteristics (including the cancer type/stage – wherever reported), depression assessment method(s), intervention type, and the main depression-related findings. Where details were not reported in the extracted dataset (e.g., the cancer stage/type, outcome instrument, timing of assessments, randomisation procedures), these were recorded as ‘not reported’ (*NR*) and were treated as ‘unclear’ within risk-of-bias judgements.

**Table 1:** Summary of studies included in the systematic review (n=20)

Article author, year	Study design	Study aim	Sample size and cancer type	Depression assessment methods	Psychological intervention type	Main results
Marco J.H., et al., 2024 [18]	Randomized controlled trial	To analyze the differential efficacy of MCP, compared to CBT in participants with cancer.	76 patients with stage I–III breast, gynaecological, prostate, lung cancer, sarcoma, lymphoma or melanoma	ODSIS	Meaning centred group psychotherapy (MCP) and cognitive behavioural therapy (CBT)	MCP and CBT was equally statistically significantly effective in improving depression.
Blanco C., et al., 2019 [19]	Randomized trial	To evaluate the efficacy of different psychotherapies for depressed women with breast cancer.	134 patients with stage I–IV breast cancer	HAM-D; BDI	Interpersonal psychotherapy; problem solving therapy (PST) and brief supportive psychotherapy (BSP)	All three psychotherapies were associated with statistically significant similar improvements in depression.
Frangou E., et al., 2021 [20]	Randomized controlled trial	To assess the impact of psychological intervention on depression and FOP in women with ovarian cancer.	107 patients with stage I–IV ovarian cancer	PHQ-9	3 90-min face to face sessions of CBT with elements of mindfulness and ACT (acceptance and commitment therapy)	PHQ-9 score improved in both intervention and control groups leaving the effect of intervention not statistically significant.
Yang Y., et al., 2022 [21]	Randomized controlled trial	To assess the efficacy of cCBT for psychological outcomes and QOL in patients with laryngectomy.	80 patients with laryngectomy due to advanced stage laryngeal carcinoma	PHQ-9	cCBT (computer-assisted cognitive behavioural therapy)	Statistically significant improvement in symptoms of depression by decreasing PHQ-9 score in cCBT group compared to control group.
Park S., et al., 2020 [22]	Randomized controlled trial	To examine the effectiveness of MBCT for psychological distress, FCR, fatigue, spiritual well-being, and QOL in Japanese ambulatory patients with Stage I–III breast cancer.	74 patients with stage I–III breast cancer	HADS	MBIs (mindfulness-based interventions)	MBCT group showed significantly better outcomes in psychological distress compared to control group.
Igelström H., et al., 2023 [23]	Randomized controlled trial	To evaluate the long-term effects of the intervention 18 and 24 months after randomization of cancer patients.	245 patients with stage I–IV breast, colorectal or prostate cancer	HADS	Internet-based stepped care intervention iCAN-DO	Symptoms of depression decreased significantly in the iCAN-DO group from baseline to 18 months but not 24 months compared to control group.
Zhang Z., et al., 2025 [24]	Randomized controlled trial	To compare rates of change in depression/anxiety and assess stigma mediation of MBSR effects.	110 patients with stage I–IV head and neck cancer	HADS	MBSR (mindfulness-based stress reduction)	MBSR significantly reduced the severity of depression symptoms in comparison to control group.
Harman Özdoğan M., et al., 2022 [25]	Randomized controlled trial	To examine effects of online MBSR combined with music therapy on stress, depression, and psychological well-being.	120 patients with cancer (type/stage NR)	BDI	MBSR combined with music therapy	Intervention group showed significantly lower stress and depression scores and higher psychological well-being than controls.
Abd Wahid N.H., et al., 2025 [26]	Experimental study (prospectively used randomization)	To evaluate depressive symptoms in newly diagnosed BC undergoing mastectomy and assess effectiveness of group CBT.	70 patients with stage I–III breast cancer who underwent surgical mastectomy	PHQ-9	G-CBT (group cognitive behavioural therapy)	G-CBT significantly reduced depressive symptoms, and the effect sustains until third month follow-up.
Shi Y., et al., 2020 [27]	Randomized controlled trial	To evaluate nurse-led positive psychology effects on sexual function, depression and subjective well-being after cervical cancer surgery.	91 patients with stage IA1–IIA2 cervical cancer post-hysterectomy	SDS (self-rating depression scale)	45–60 min interventions twice a week for 4 weeks	At 3 and 6 months, depression and well-being improvements were statistically significantly higher in intervention vs control.
Do M.T., et al., 2024 [28]	Non-randomized controlled intervention study	To evaluate effectiveness of group-based psychotherapy in NSCLC with depression in Vietnam (COVID-19 era).	40 patients with stage I–IV non-small cell lung carcinoma	PHQ-9	Online group-based psychotherapy	Unclear differences in general depression between intervention and control groups.
Chu X., et al., 2020 [29]	Randomized controlled trial (method of randomization not reported)	To study effect of mindfulness-based CBT on mental health and quality of life in breast cancer.	84 patients with stage 0–III breast cancer	HADS	MBCT (mindfulness-based cognitive therapy)	Mental health outcomes improved at week 8 and remained significant at week 12 vs. controls.
Jeitler M., et al., 2017 [30]	Prospective cohort study	To evaluate the effectiveness of an integrative day-care clinic program based on mind–body medicine and lifestyle modification for cancer patients	86 patients with breast cancer (stage NR)	HADS	Program focusing on mind-body techniques and health-promoting lifestyle modification	Significant improvements in anxiety and depression scores, fatigue, and quality of life compared with waiting group; improvements persisted at 6-month follow up.
Kenne Sarenmalm E., et al., 2017 [31]	3-month follow up study of RTC	To determine efficacy of MBSR for mood disorders in women with breast cancer.	166 patients with early-stage breast cancer	HADS	MBSR; self-instructing MBSR	Women in the MBSR group experienced significant depression improvements vs non-MBSR.
Lee C., et al., 2017 [32]	Prospective cohort study	To investigate effectiveness of an MBSR programme in metastatic breast cancer.	18 patients with metastatic breast cancer	HADS and additional psychological distress/quality-of-life scales	MBSR	Significant reductions in anxiety and depressive symptoms and improvements in quality of life and stress perception following the intervention
Mirmahmoodi M., et al., 2020 [33]	Randomized controlled trial	To investigate effectiveness of MBSR group counselling on anxiety, depression, stress, and laboratory tests (cortisol, CRP).	44 patients with breast cancer without metastasis	BDI	MBSR group counselling	No significant difference between MBSR and control in reduction of perceived stress and depression.
Shao D., et al., 2021 [34]	Randomized controlled trial	To investigate guided self-help MBI effects on depression, anxiety, sleep disorders and mechanisms.	144 patients with stage I–IV breast cancer	PHQ-9	Guided self-help MBI	Depression and sleep disorder symptoms improved vs wait-list; maintained at a 1- and 3-month follow-up.
Sheikhzadeh M., et al., 2021 [35]	Randomized controlled trial	To compare efficacy of MBCT and CBT for anxiety, depression, and fatigue.	60 cancer patients (type and stage not specified)	BDI	MBCT; CBT	Depression, anxiety, fatigue reduced in both MBCT and CBT vs control; no significant difference between MBCT and CBT.
Janusek L., et al., 2019 [36]	Randomized controlled trial	To determine whether MBSR benefits psychological, behavioural, and immunological function in women recently diagnosed with breast cancer.	192 patients with early-stage breast cancer	NR	MBSR	MBSR showed decreasing trajectories of perceived stress, fatigue, sleep disturbance, and depressive symptoms.
Wang H., et al., 2023 [37]	Observational pilot study	To investigate MBSR effects on anxiety and depression during postoperative chemotherapy.	225 patients (cancer type/stage NR in extracted data)	SDS (self-rating depression scale)	MBSR	SDS and SAS improved in the MBSR group compared with controls.

The 20 included studies in the supplied order were: [18]–[37]. Specifically: [18] compared meaning-centred psychotherapy with CBT in a mixed cancer cohort; [19] compared three psychotherapy modalities in depressed women with breast cancer; [20] evaluated a brief CBT-based intervention (with mindfulness/ACT elements) in ovarian cancer; [21] tested computer-assisted CBT after laryngectomy; [22] evaluated MBCT in stage I–III breast cancer; [23] examined an internet stepped-care-intervention with a long follow-up; [24] tested MBSR in head and neck cancer; [25] evaluated online MBSR combined with music therapy; [26] assessed group CBT in breast cancer post-mastectomy; [27] tested a nurse-led positive psychology intervention in cervical cancer survivors; [28] evaluated online group psychotherapy for NSCLC with depression; [29] tested MBCT in breast cancer with a follow-up up to week 12; [30] described a mind–body and lifestyle programme; [31] evaluated MBSR and self-instructing MBSR in early-stage breast cancer; [32] reported an MBSR programme in metastatic breast cancer (with key details NR); [33] evaluated MBSR group counselling in non-metastatic breast cancer; [34] tested guided self-help mindfulness in breast cancer; [35] compared MBCT and CBT (cancer type/stage NR); [36] evaluated MBSR in newly diagnosed early-stage breast cancer (depression instrument NR); and [37] reported an observational pilot of MBSR during postoperative chemotherapy.

The total participants across all studies were n=2,166. Cancer populations were heterogeneous, dominated by breast cancer cohorts (early stage, metastatic, postoperative and chemotherapy settings) [19], [22], [26], [29], [31]–[34], [36], [37], with additional cohorts in ovarian cancer [20], cervical cancer [27], head and neck cancer [24], NSCLC [28], post laryngectomy laryngeal carcinoma [21], and mixed cancer cohorts [18], [23], [35]. Study designs were predominantly randomised, though nonrandomised and observational designs were also included [28], [30], [32], [37].

### Risk of bias

Overall, most RCTs were judged as having some concerns of bias (**Table 2**), primarily due to unclear randomization procedures and reliance on self-reported outcomes. Non-randomized studies demonstrated a serious risk of bias related to confounding and selection bias.

**Table 2:** Risk of bias assessment of included studies

Study (Author, Year)	Design	Tool	Key domain-level concerns	Overall risk of bias
Marco, 2024 [18]	Randomised trial	RoB 2	Unblinded participants with self-reported outcome; handling of missing outcome data unclear; selective reporting unclear	Some concerns
Blanco, 2019 [19]	Randomised trial	RoB 2	Randomisation/allocation concealment insufficiently described; blinding of outcome assessment unclear; missing data handling unclear	Some concerns
Frangou, 2021 [20]	Randomised trial	RoB 2	Randomisation/allocation concealment unclear; self-reported outcome in an unblinded setting; missing data handling unclear	Some concerns
Yang, 2022 [21]	Randomised trial	RoB 2	Unblinded participants with self-reported outcome; randomisation process insufficiently described; missing data handling unclear	Some concerns
Park, 2020 [22]	Randomised trial	RoB 2	Self-reported outcome in an unblinded setting; randomisation process unclear; missing data handling unclear	Some concerns
Igelström, 2023 [23]	Randomised trial	RoB 2	Self-reported outcome in an unblinded setting; attrition risk increased by long follow-up; reporting details unclear	Some concerns
Zhang, 2025 [24]	Randomised trial	RoB 2	Self-reported outcome in an unblinded setting; randomisation process unclear; selective reporting concerns because prespecification of mediation analyses was unclear	Some concerns
Harman Özdoğan, 2022 [25]	Randomised trial	RoB 2	Randomisation process insufficiently described; self-reported outcome in an unblinded setting; missing data handling unclear	Some concerns
Abd Wahid, 2025 [26]	Randomised trial	RoB 2	Randomisation stated but method not described; self-reported outcome in an unblinded setting; missing data handling unclear	Some concerns
Shi, 2020 [27]	Randomised trial	RoB 2	Randomisation process unclear; outcome reporting based on numbers improved may increase reporting bias; self-reported outcome	Some concerns
Do, 2024 [28]	Non-randomised controlled study	ROBINS-I	Confounding and selection bias likely; intervention classification/comparator definition unclear; self-reported outcome in an unblinded setting	Serious
Chu, 2020 [29]	Randomised trial	RoB 2	Randomisation process not reported; self-reported outcome in an unblinded setting; missing data handling unclear	Some concerns
Jeitler, 2017 [30]	Non-randomised intervention study	ROBINS-I	Confounding and selection bias likely; comparator structure and intervention classification insufficiently described; outcome reporting incomplete in extracted data	Serious
Kenne Sarenmalm, 2017 [31]	Randomised trial	RoB 2	Randomisation process insufficiently described; self-reported outcome in an unblinded setting; missing data handling unclear	Some concerns
Lee, 2017 [32]	Non-randomised pilot intervention study	ROBINS-I	High risk of confounding and selection bias; outcome measurement/reporting insufficiently described in extracted data; exceedingly small sample	Serious
Mirmahmoodi, 2020 [33]	Randomised trial	RoB 2	Small sample; randomisation process unclear; self-reported outcome in an unblinded setting; missing data handling unclear	Some concerns
Shao, 2021 [34]	Randomised trial	RoB 2	Self-reported outcome in an unblinded setting; wait-list control may increase risk of deviations from intended interventions; missing data handling unclear	Some concerns
Sheikhzadeh, 2021 [35]	Randomised trial	RoB 2	Randomisation process insufficiently described; self-reported outcome in an unblinded setting; missing data handling unclear	Some concerns
Janusek, 2019 [36]	Randomised trial	RoB 2	Outcome measurement/reporting insufficiently described in extracted data; randomisation process unclear; selective reporting unclear	Some concerns
Wang, 2023 [37]	Observational pilot study	ROBINS-I	Confounding and selection bias likely; uncontrolled pilot design/comparator limitations; self-reported outcome	Serious

### Outcome measures

Depression symptoms were assessed by using multiple validated instruments (**Table 3**), dominated by HADS (6 studies) [22], [23], [24], [29], [30], [31], and PHQ9 (5 studies) [20], [21], [26], [28], [34]. BDI was used in three studies [25], [33], [35], SDS in two [27], [37], and single studies used HAM-D (with BDI) [19] or ODSIS [18]. Two studies did not specify the depression instrument within the extracted dataset [32], [36]. This heterogeneity underpinned the decision to synthesise outcomes narratively rather than pool the effect sizes.

**Table 3:** Depression assessment scales used across the included studies

Depression scale	Studies	Notes
ODSIS	[18]	Self-report measure capturing depression-related impairment and severity.
HAM-D; BDI	[19]	Clinician-rated plus self-report in a cohort explicitly described as depressed.
PHQ-9	[20], [21], [26], [28], [34]	Self-report; used across post-treatment, surgical/perioperative, and online delivery contexts.
HADS	[22], [23], [24], [29], [30], [31]	Self-report; often interpreted as distress, including depression subscale/total score.
BDI	[25], [33], [35]	Self-report; used in mindfulness and psychotherapy comparison designs.
SDS	[27], [37]	Self-report; also reported as ‘number improved’ in at least one study [27].
NR / not specified in extracted data	[32], [36]	Depression instrument not captured in the supplied extraction dataset.

### Psychological interventions and depressive symptoms

As pooled estimates were not available from the extraction dataset and depression outcomes varied by measure and analytic reporting, findings are summarised as a narrative synthesis across intervention types and clinical contexts.

In a mixed cancer cohort, meaning-centred psychotherapy and CBT were both associated with statistically significant improvements in depression, with no differential efficacy between the arms [18]. In depressed women with breast cancer, interpersonal psychotherapy, problem-solving therapy, and brief supportive psychotherapy each produced statistically significant improvements in depression, with similar effects across the modalities [19]. A brief CBT intervention including mindfulness/ACT elements in ovarian cancer was associated with PHQ-9 improvement in both intervention and control groups, with a statistically insignificant between-group effect on depression [20]. In contrast, computer-assisted CBT after laryngectomy was associated with statistically significant improvement in depressive symptoms compared with control (PHQ-9 reduction) [21]. In stage I–III breast cancer, MBCT produced significantly better outcomes in psychological distress compared with controls (HADS-based outcomes) [22]. An internet-based stepped care programme reduced depression from the baseline to 18 months but not at 24 months compared with controls [23]. MBSR reduced the depressive symptom severity compared with treatment as usual in stage I–IV head and neck cancer (HADS), with accompanying mediation analysis involving internalised stigma [24]. Online MBSR combined with music therapy reduced depression (BDI) and stress, and improved psychological well-being compared with controls, although cancer type and stage were not specified in the extraction dataset [25]. Group CBT in patients undergoing mastectomy for stage I–III breast cancer significantly reduced PHQ-9 depressive symptoms, with maintenance of effect to the third-month follow-up [26]. A nurse-led positive psychology intervention in early-stage cervical cancer survivors demonstrated statistically significant improvements in depression and well-being at 3 and 6 months compared with usual care (SDS) [27]. A pilot non-randomised trial of online group psychotherapy for NSCLC patients with depression reported unclear differences in depression outcomes between the intervention and control groups [28]. Another MBCT study reported improvements in mental health outcomes by week 8, with between-group differences persisting at week 12 (HADS) [29]. Two studies relevant to mind–body/MBSR approaches had key depression outcome details that were not extractable from the dataset [30], [32]. In early-stage breast cancer, an MBSR trial (with a self-instructing MBSR arm) reported significant improvement in depression scores versus nonMBSR- comparators (HADS) [31]. Conversely, MBSR group counselling in non-metastatic breast cancer found no significant between-group reduction in depression or perceived stress (BDI) [33]. Guided self-help mindfulness interventions produced significant improvements in depression (PHQ-9), with maintenance at a 1- and 3-month follow-up compared with wait-list controls [34]. In a further direct comparison, MBCT and CBT both reduced depression relative to control, with no significant between-group difference between MBCT and CBT [35]. A further MBSR trial in newly diagnosed early-stage breast cancer reported favourable trajectories in depressive symptoms (instrument NR in the extraction dataset) alongside improvements in stress/behavioural and immunological domains [36]. An observational pilot study during postoperative chemotherapy reported improvement in depression (SDS) within an MBSR programme compared with controls, though the non-randomised design limits causal inference [37].

Overall, across the intervention families, the direction of effect most often favoured intervention over control for depressive symptoms, but multiple studies reported null between the group effects or incomplete reporting, and findings varied by intervention intensity, delivery mode, and population [18]– [37].

### Depression assessment instruments

Depression assessment was heterogeneous, limiting cross study comparability without pooling. The most frequently used instruments were HADS [22], [23], [24], [29], [30], [31], and PHQ9 [20], [21], [26], [28], [34]. BDI was used in several mindfulness and comparative psychotherapy studies [25], [33], [35], SDS was used in the positive psychology RCT and an observational chemotherapy pilot study [27], [37], and single studies used HAMD/BDI (paired) [19] or ODSIS [18]. Two studies did not report the depression instrument within the extracted dataset [32], [36]. Across studies, the use of both clinician-rated (HAMD) and self-report scales (e.g., PHQ9, HADS, BDI, SDS, ODSIS) implies differences in sensitivity to change and susceptibility to the expectancy and reporting effects.

### Depression prevalence among cancer patients

The included evidence base is not designed to estimate the population level prevalence of depression in oncology, because most included studies are intervention trials (often with symptom/distress inclusion criteria) rather than epidemiological cohorts [18]–[37]. Nevertheless, the study samples demonstrate that a clinically relevant depressive symptom burden is common enough to support repeated trial recruitment across cancer settings. Some studies explicitly enrolled participants with depression or significant depressive symptoms (e.g., depressed women with breast cancer in a psychotherapy trial [19]; NSCLC patients with depression in a pilot intervention study [28]), which represents enriched samples and cannot be interpreted as prevalence in the underlying oncology population. Where trials enrolled broader oncology cohorts (commonly, breast cancer) without explicit depression case inclusion, depressive symptoms were typically evaluated as continuous outcomes rather than as prevalence estimates [18], [22], [23], [31], [36], [37].

## Discussion

### Principal interpretation

Across head-to-head psychotherapies and CBT-family interventions, most trials reported significant improvements in depressive symptoms, while consistent superiority of any single modality was uncommon [18–21]. Mindfulness-family interventions and stepped digital care similarly tended to favour intervention over control, but null findings occurred and durability was inconsistent, particularly where clinical characterisation or delivery intensity was limited [22–25].

### Symptom-guided intervention

A pragmatic, symptom-guided approach may improve intervention ‘fit’ (**Table 4**). For post-mastectomy adjustment with behavioural withdrawal and low mood, group CBT showed sustained PHQ-9 improvement to three months [26]. In survivorship contexts where depressive symptoms co-occur with reduced well-being (including sexual health burden), nurse-led positive psychology improved depression at three and six months [27]. Where access constraints necessitate remote delivery, non-specialist online group psychotherapy showed uncertain between-group benefit in depressed NSCLC, supporting the need for engagement scaffolding and stepped escalation [28]. For rumination- and worry-dominant distress, MBCT showed short-term benefit on HADS-based outcomes [29]. Evidence for broader mind–body/lifestyle programmes was incomplete (NR for key depression effects), limiting inference [30]. For early-stage breast cancer with mood disturbance, structured MBSR approaches improved HADS-depression in some trials, whereas evidence in metastatic disease remains sparse and under-reported (NR) [31,32]. Divergent MBSR findings (including null effects) highlight probable moderation by baseline severity, dose, and adherence [33]. Low-intensity guided self-help mindfulness can reduce PHQ-9 depression with maintenance to three months and may suit patients with limited capacity for frequent appointments [34]. When fatigue is prominent alongside low mood, CBT and MBCT appear similarly effective for depression, suggesting symptom-cluster priorities can guide modality choice [35]. Some trials also reported improvements in stress-behavioural and immune-related trajectories alongside depressive symptom change, but observational designs during chemotherapy leave residual confounding [36,37].

**Table 4:** Symptom-guided selection of psychological intervention families for depressive symptoms in oncology

Intervention family	Typical patient presentation	Key supporting studies (citation numbers)	Delivery mode	Expected effect on depression	Notes on feasibility
Structured psychotherapy (IPT/PST/BSP)	Probable MDD; interpersonal stressors; needs clinician-delivered therapy	19, 39	Mostly therapist-led (NR in extraction)	Improves symptoms; no clear modality superiority	Resource-intensive; requires trained workforce
Meaning-/dignity-centred	Demoralisation, loss of meaning, existential distress	18	Group/individual (NR)	Improves depression and meaning-related outcomes	Often fits palliative/supportive care pathways
CBT-family (incl. cCBT, group CBT)	Behavioural withdrawal; peri-operative/surgical stress; fatigue-linked cognitions	20–21, 26, 35, 38, 46	In-person or digital	Typically improves depression; may help symptom clusters	Scalable; group/digital formats need adherence supports
Mindfulness-family (MBCT/MBSR/guided)	Rumination/worry; distress reactivity; stigma-related self-judgement	22, 24–25, 29, 31, 33–34, 36–37	Group/online/self-help	Often improves depression; some null trials	Engagement and “dose” likely moderate outcomes
Low-intensity / digital stepped care	Access barriers; limited capacity; preference for remote support	23, 28, 34, 45	Online, stepped escalation	Modest, variable; better with support	Usage predicts effect; needs onboarding and monitoring
Supportive/adjunctive (positive psychology, hypnosis, music)	Survivorship well-being focus; co-occurring QoL burdens	27, 47, 49	Nurse-led/group/supportive care	Modest improvements; adjunctive role	Pragmatic to embed in oncology services; evidence mixed

### Contextualisation with external evidence

Broader depression evidence supports scalability: behavioural activation delivered by less specialised staff was non-inferior to CBT and more cost-effective, aligning with task-sharing logic in oncology [38]. Cancer-specific syntheses and observational work indicate that depression correlates and needs vary by cancer site and treatment context, and comparative efficacy across psychotherapies is often modest, thus reinforcing a matched-care rather than a one-size-fits-all model [39–44]. Digital and blended approaches are feasible, but engagement is a key constraint; usage/adherence predicts outcome, and adjunctive modalities (e.g., hypnosis, dignity-focused work, music-based supportive care) may be appropriate for selected depressive presentations, though the trial quality and sample sizes remain limiting [45–49].

### Limitations and implications

This review is limited by substantial heterogeneity in cancer populations, intervention dose/delivery, and depression instruments (including NR in some studies), thus precluding meta-analysis and complicating cross-study comparability. Risk of bias was frequently driven by incomplete reporting of randomisation/allocation, unblinded self-report outcomes, and non-randomised designs.

## Conclusion

This systematic review shows that depressive symptoms are common among cancer patients and are associated with poorer psychological outcomes in prospective cohorts. Although psychosocial interventions consistently reduce the depressive symptom severity and improve the quality of life, current evidence does not clearly demonstrate direct effects on depression progression.

From a clinical perspective, these findings support routine screening for depressive symptoms while using validated instruments and timely referral to the appropriate psychosocial care. Integration of psychological support into oncology care pathways may improve patient well-being and could indirectly influence outcomes through better treatment adherence and patient engagement.

Future research should prioritise well-powered randomized controlled trials which also needed to clarify biological mechanisms linking depression with cancer progression and to evaluate scalable models of care, such as tele-based and collaborative psychosocial interventions. Standardised approaches to screening, intervention delivery, and outcome reporting will be important for strengthening the evidence base and improving the integration of mental health care in oncology.

## Abbreviations

ACT – Acceptance and Commitment Therapy

BDI – Beck Depression Inventory

CBT – Cognitive Behavioural Therapy

cCBT – Computer-assisted Cognitive Behavioural Therapy

FOP – Fear of Progression

HADS – Hospital Anxiety and Depression Scale

MBCT – Mindfulness-Based Cognitive Therapy

MBI – Mindfulness-Based Intervention

MBSR – Mindfulness-Based Stress Reduction

PHQ-9 – Patient Health Questionnaire-9

QoL – Quality of Life

RCT – Randomized Controlled Trial

RoB – Risk of Bias

SDS – Self-Rating Depression Scale
